# New Sensitive Kinetic Spectrophotometric Methods for Determination of Omeprazole in Dosage Forms

**DOI:** 10.1155/2009/307045

**Published:** 2009-12-02

**Authors:** Ashraf M. Mahmoud

**Affiliations:** Department of Pharmaceutical Analytical Chemistry, Faculty of Pharmacy, Assiut University, Assiut 71256, Egypt

## Abstract

New rapid, sensitive, and accurate kinetic spectrophotometric methods were developed, for the first time, to determine omeprazole (OMZ) in its dosage forms. The methods were based on the formation of charge-transfer complexes with both iodine and 2,3-dichloro-5,6-dicyano-1,4-benzoquinone (DDQ). The variables that affected the reactions were carefully studied and optimized. The formed complexes and the site of interaction were examined by UV/VIS, IR, and ^1^H-NMR techniques, and computational molecular modeling. Under optimum conditions, the stoichiometry of the reactions between OMZ and the acceptors was found to be 1 : 1. The order of the reactions and the specific rate constants were determined. The thermodynamics of the complexes were computed and the mechanism of the reactions was postulated. The initial rate and fixed time methods were utilized for the determination of OMZ concentrations. The linear ranges for the proposed methods were 0.10–3.00 and 0.50–25.00 *μ*g mL^−1^ with the lowest LOD of 0.03 and 0.14 *μ*g mL^−1^ for iodine and DDQ, respectively. Analytical performance of the methods was statistically validated; RSD was <1.25% for the precision and <1.95% for the accuracy. The proposed methods were successfully applied to the analysis of OMZ in its dosage forms; the recovery was 98.91–100.32% ± 0.94–1.84, and was found to be comparable with that of reference method.

## 1. Introduction

Omeprazole (OMZ), 5-methoxy-2-[[(4-methoxy-3,5-dimethyl-2-pyridinyl)methyl]sulphinyl]-1H-benzimidazole, is the first member of the “proton pump inhibitors” that are widely used for the prophylaxis and treatment of both gastro-duodenal ulcers and symptomatic gastrooesophageal reflux [1]. Also, it is highly effective in the treatment of Zollinger-Ellison syndrome [1, 2]. 


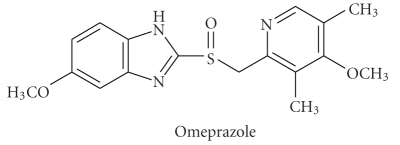


The therapeutic importance of OMZ was behind the development of many analytical methods for its determination in the pharmaceutical formulations and/or biological samples. These methods include spectrophotometry [3–13], electrochemical methods [14], HPLC [15–17], and electrophoresis [18]. As compared to the electrochemical, electrophoretic, and chromatographic methods, despite of their higher sensitivity, the spectrophotometric methods are more versatile and easy to apply. Direct UV spectrophotometry has been proved to be inaccurate due to the matrix interference [3]. Some reported visible spectrophotometric methods [4], being based on the nonselective oxidation of OMZ, may be also influenced by the excipients resulting in inaccurate results. Therefore, accurate visible spectrophotometric methods are still required for the determination of OMZ in quality control laboratories. The molecular interactions between the electron donating pharmaceutical compounds [19–23] and the electron acceptors are generally associated with the formation of intensely colored charge-transfer (CT) complexes [24]. Molecular CT complexes of OMZ with both iodine as *σ*-acceptor and 2,3-dichloro-5,6-dicyano-1,4-benzoquinone (DDQ) as *π*-acceptor have not been studied yet. Therefore, both acceptors were selected as analytical reagents in the present study. Kinetic spectrophotometric methods are becoming of great interest in pharmaceutical analysis [25] as they offered some advantages such as improved selectivity, avoiding the interference of the colored and/or turbidity background of the samples, and possible avoiding of the interference of the other active compounds present in the commercial product if they are resisting the reaction conditions established for the proposed kinetic method. Therefore, the development of new kinetic spectrophotometric methods for the determination of OMZ in its pharmaceutical preparations was targeted. Studying the kinetics, molecularities, thermodynamics, and association constants of the CT complexes as well as their examination by the spectroscopic techniques, and computational molecular modeling usually provide information about the nature and mechanism of the CT complex formation. An important application for CT complexes with iodine is that the antithyroid activity could be expected for drugs whose association constant with iodine exceeds 100 l.mol^−1^. Above this value the antithyroid activity is directly related to the value of the association constant [26]. Therefore, it was important to determine the association constant for OMZ-iodine complex beside the method development.

## 2. Experimental

### 2.1. Apparatus

A Shimadzu model UV-1601 PC (Japan) UV-VIS double beam spectrophotometer with matched 1-cm quartz cells was used for recording the electronic absorption spectra and all measurements. ^1^H-NMR spectra were recorded in DMSO-d_6_ at 500 MHz by Bruker-Ultra Shield instrument (Bruker Co., USA). IR spectra were recorded by FT-IR model Spectrum BX spectrometer (Perkin-Elmer, USA).

### 2.2. Chemicals, Reagents, and Pharmaceutical Formulations

OMZ (Hetero Drugs Ltd, Hyderabad, India) was used as working standard. Iodine, resublimed (Riedel-De-Haen AG, Germany), was 2 mg mL^−1^ in 1,2-dichloroethane. The solution was stable for at least 1 week at 4°C. 2,3-Dichloro-5,6-dicyano-1,4-benzoquinone (DDQ; Merck, Schuchardt, Munich, Germany) solution was 4 mg mL^−1^ in acetonitrile, and it was freshly prepared daily. Gastrazole capsules (Riyadh Pharma, Saudi Arabia) and Losec tablets (Astra Zeneca, Sweden) were labeled to contain 20 mg OMZ per capsule or tablet. All solvents and other chemicals used throughout this study were of analytical grade.

### 2.3. Preparation of Standard and Sample Solutions

#### 2.3.1. Preparation of Stock Standard Solution

Into a 100 mL calibrated flask, 25 mg of OMZ was accurately weighed, dissolved in 20 mL 1,2-dichloroethane (for the reaction with iodine) or acetonitrile (for the reaction with DDQ), and completed to volume with the same solvent to obtain a stock solution of 0.25 mg mL^ −1^. These stock solutions were further diluted with the respective solvents to obtain suitable concentrations that lie in the linear range of each particular assay method.

#### 2.3.2. Preparation of Tablets or Capsules Solution

Twenty tablets or the contents of twenty capsules of each formulation were weighed and finely powdered. A quantity of the powder equivalent to 25.00 mg of OMZ was transferred into a 100 mL calibrated flask, dissolved in 20 mL of the appropriate solvent, swirled and sonicated for 5 minutes. The flask was completed to volume with the same solvent, shaken well for 15 minutes, and filtered. The first portion of the filtrate was rejected and a measured volume of the filtrate was diluted quantitatively with the respective solvent to yield suitable concentrations that lie in the linear range of each particular assay method.

### 2.4. General Analytical Procedure

#### 2.4.1. Initial Rate Method

Accurately measured aliquots of OMZ solution (1.00–250.00 *μ*g mL^−1^) were transferred into separate 10 mL calibrated flasks. One milliliter of iodine (2.0 mg mL^−1^) or DDQ (4.0 mg mL^−1^) was added. The solution was diluted to volume with 1,2-dichloroethane (for iodine) or acetonitrile (for DDQ) and mixed well. After mixing, the reaction mixture was monitored at room temperature (25 ± 2°C) and the absorbance was recorded as a function of time for 45 and 5 minutes at 362 or 418 for iodine and DDQ, respectively, against reagent blank treated similarly.

#### 2.4.2. Fixed Time Method

One milliliter of the standard or sample solution of OMZ (1.00–250.00 *μ*g mL^−1^) was transferred into 10 mL calibrated flasks. One milliliter of iodine (2.0 mg mL^−1^) or DDQ (4.0 mg mL^−1^) was added. The solution was diluted to volume with the appropriate solvent and mixed well. The reaction was allowed to proceed for 45 and 5 minutes at room temperature (25 ± 2°C) for iodine and DDQ, respectively. The absorbance of the resulting solutions was measured at 362 or 418 for iodine and DDQ, respectively, against reagent blanks treated similarly.

### 2.5. Determination of the Stoichiometry of CT Reactions

Job's method of continuous variation [27] was employed. Master equimolar solutions of OMZ and acceptors were prepared. The concentrations of these solutions were 1 × 10^−5^ M (for iodine) in 1,2-dichloroethane and 1 × 10^−4^ M (for DDQ) in acetonitrile. Series of 10 mL portions of the master solutions of OMZ and the acceptor were made up in 10 mL calibrated flasks comprising different complementary proportions (0 : 10, 1 : 9,…, 9 : 1, 10 : 0). The reactions were further manipulated according to the general analytical procedures.

### 2.6. Preparation of the Complexes for IR and ^1^H-NMR Measurements

A quantity of 0.1 mmol (~35 mg) of OMZ, dissolved in the appropriate solvent, was added to an equimolar amount of the acceptor in the same solvent in a round-bottom flask containing ~30 mL of the respective solvent and stirred for 30 minutes. The solvents were evaporated under reduced pressure, and the resulting residues were dried over calcium chloride. The dried residues were used for both IR and ^1^H-NMR measurements.

### 2.7. Association Constant and Free Energy Change

Series of OMZ solutions (0.4 × 10^−4^–2.1 × 10^−3^ M) in the appropriate solvent were prepared. In addition to these solutions, iodine (3 × 10^−3^ M) and DDQ (3.6 × 10^−3^ M) solutions in the appropriate solvent were equilibrated for 30 minutes in a thermostatically controlled water bath at 25 ± 2°C. Five milliliters of each acceptor solution was mixed rapidly with 5 mL of OMZ solution in 10 mL calibrated flasks. The absorbance of the solutions was measured immediately at the corresponding maxima against reagent blanks treated similarly.

### 2.8. Data Acquisition and Processing

The kinetic data recorded for the proposed methods was transformed to the Slide Write Plus software, version 5.011 (Advanced Graphics Software, Inc., CA, USA) for curve fitting, regression analysis, and statistical calculations. The initial rate (*V*) of the reaction at different OMZ concentrations was obtained from the slope of the tangent of the absorbance-time curve. The calibration curve was constructed by plotting the logarithm of the initial rate of the reaction (log *V*) versus the logarithm of the molar concentration of OMZ (log *C*). Alternatively, the calibration curve was constructed by plotting the absorbance measured after a preselected fixed time versus the concentration of OMZ. The limits of detection (LOD) and limits of quantitation (LOQ) were determined [28] using the formula: LOD or LOQ  = *κ*SD_*a*_/*b*, where *κ* = 3.3 for LOD and 10 for LOQ, SD_a_ is the standard deviation of the intercept, and *b* is the slope.

## 3. Results and Discussion

### 3.1. Spectral Characteristics of the Charge-Transfer Reactions

#### 3.1.1. Reaction with *σ*-Acceptor

**Figure 1:**
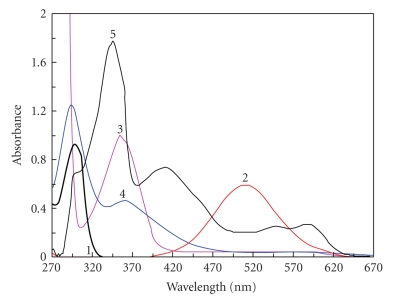
Absorption spectra of OMZ (1), iodine, 0.20 mg mL^−1^ in 1,2-dichloroethane (2), DDQ, 0.40 mg mL^−1^ in acetonitrile (3), OMZ-iodine complex in 1,2-dichloroethane at 25 ± 2°C (4), and OMZ-DDQ complex in acetonitrile at 25 ± 2°C (5). Concentrations of OMZ were 30.00, 1.50, and 20.00 *μ*g mL^−1^ for the absorption spectra numbers (1), (4), and (5), respectively.

**Scheme 1:**
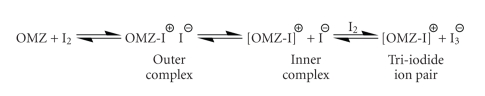


The violet-colored iodine solution in 1,2-dichloroethane was changed into lemon yellow upon addition of OMZ. Examination of the absorption spectrum of OMZ-iodine reaction product showed two absorption maxima at 290 and 362 nm; the first peak is about twofold more intense than the second (Figure 1). The spectrum was found to be identical with that of tri-iodide ion (I_3_^−^) in 1,2-dichloroethane. This identity in both spectra proved that the color change and the appearance of the new bands at 290 and 362 nm were attributed to the formation of OMZ-iodine CT complex with an ionized structure OMZ-I^+^ ⋯ I_3_^−^. This OMZ complex should originate from an early intermediate outer complex as explained by Scheme 1. Confirming the CT nature of the reaction, the violet-colored solution of iodine in 1,2-dichloroethane was restored upon extracting OMZ from the complex by shaking with aqueous mineral acids. Measurements were carried out at 362 nm to avoid the interference from the native UV absorption of OMZ at 290 nm.

#### 3.1.2. Reaction with DDQ

**Scheme 2:**
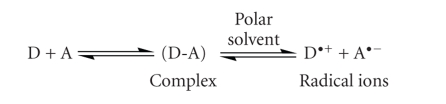


The interaction of OMZ with DDQ in nonpolar solvents such as chloroform led to formation of colored CT complexes with low *ε*-values. However, in acetonitrile OMZ-DDQ CT complex with high *ε*-values was formed. This was attributed to complete electron transfer from OMZ (D) to the acceptor moiety (A) accompanied by the formation of intensely colored radical ions in polar solvents [20], according to Scheme 2.

**Figure 2:**
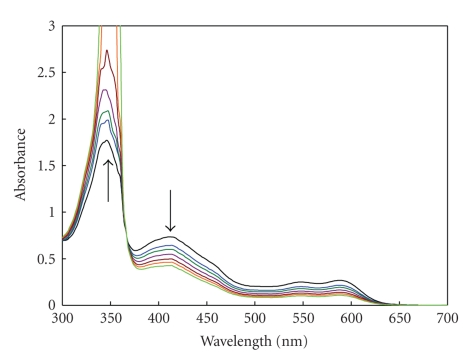
Absorption spectra of OMZ-DDQ complex as a function of time. The complex was generated by carrying out the reaction of OMZ (20.00 *μ*g mL^−1^) with DDQ (0.40 *μ*g mL^−1^) in acetonitrile at 25 ± 2°C.

The high ionizing power of the acetonitrile is the driving force for dissociation of the (D-A) complex to form the intense colored radical ion. As shown in Figure 1, OMZ has no considerable absorption band in the range of 400–600 nm; however, the OMZ-DDQ CT complex has some absorption bands characterized by 4 maxima at 346, 418, 548, and 588 nm presumably due to the formation of a deep red colored CT complex. The intensity of the maximum at 346 nm is about two- to threefold the maximum at 418 nm, while the intensity of the maximum at 418 nm is about 2.7-fold the maxima at both 548 and 588 nm. However, the measurements at 346 nm were irreproducible. Investigating the electronic spectrum of OMZ-DDQ complex as a function of time in acetonitrile (Figure 2) revealed that the deep red-colored solution (the intensity of the absorption bands at the 400–600 nm region) began to increase up to 5 minutes and then leveled off for about 20 minutes and finally began to disappear by increasing the time; however, the intensity of the band at 346 nm still is increasing up to more than 3 hours. Therefore, the measurements were carried out at 418 nm to obtain the higher sensitivity and to avoid the irreproducibility of the measurements at 346 nm. Such spectral features were coincident with those observed for the interaction of DDQ with other donors [21, 29, 30] and the reported values of the radical anions of DDQ obtained by the reduction method [31]. 

### 3.2. Optimization of Reaction Conditions

#### 3.2.1. Effect of Reagent Concentration

Studying the effect of the reagent concentrations on CT complex formation indicated that 1 mL of iodine (2.0 mg mL^−1^) and DDQ (4.0 mg mL^−1^) working solutions were the optimum concentrations (i.e., the final concentrations of both reagents in the measured solution become 0.2 and 0.4 mg mL^−1^, resp.). Higher concentrations of both reagents resulted in either higher blank readings or decreased absorption intensity.

#### 3.2.2. Effect of Solvent

In order to select the most appropriate solvent, the reactions were carried out in different solvents. Small shifts in the position of the maximum absorption peaks were observed, and the absorption intensities were also influenced. 1,2-Dichloroethane was found to be an ideal solvent in case of iodine, because it is favorable for the formation of tri-iodide ion pair. Methylene chloride, chloroform, and carbon tetrachloride produced lower absorption readings. Polar solvents were found to be unsuitable as their blanks with iodine gave higher readings. However, acetonitrile found to be an ideal solvent for DDQ, because it offered the maximum sensitivity. This was attributed to its high dielectric constant that promotes the maximum yield of radical anions, in addition to its high solvating power for DDQ [32].

#### 3.2.3. Effect of Reaction Temperature

The optimum reaction temperature for the investigated acceptors was determined by following the color development at different temperatures (25, 40, 50, 60, and 70°C) using fixed concentrations of OMZ and the acceptors. The results indicated that room temperature (25 ± 2°C) was the ideal selection and higher temperatures was found to decrease the absorption intensity.

#### 3.2.4. Effect of Time

**Figure 3:**
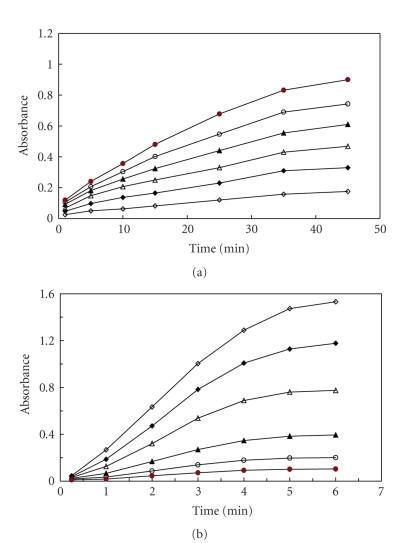
The absorbance-time curve for the reaction of OMZ at 25 ± 2°C with (a) iodine (0.20 mg mL^−1^) at 362 nm; the concentrations of OMZ were 1.45 × 10^−6^ (⋄), 2.90 × 10^−6^ (◆), 4.35 × 10^−6^ (∆), 5.80 × 10^−6^ (▴), 7.25 × 10^−6^ (°), and 8.70 × 10^−6^ M (●), and (b) DDQ (0.40 mg mL^−1^) at 418 nm; the concentrations of OMZ were 0.73 × 10^−5^ (●), 1.45 × 10^−5^ (°), 2.90 × 10^−5^ (▴), 5.80 × 10^−5^ (∆), 8.70 × 10^−5^ (◆), and 1.15 × 10^−4^ M (⋄).

Since the formation of the colored CT products increases with time, it was deemed useful to generate absorption-time curves (Figure 3) in order to determine the kinetics and thermodynamics of the investigated CT reactions. This was performed by monitoring the color development at room temperature. Complete color development was attained after 45 and 5 minutes for iodine and DDQ, respectively (Figure 3). The developed colors remained stable at room temperature for at least further 40 and 20 minutes for iodine and DDQ, respectively. The absorption intensity of OMZ-DDQ complex decreased dramatically after 20 minutes. The optimum reaction conditions for both acceptors were summarized in Table 1.

### 3.3. Stoichiometry of the CT Reaction and the Proposed Site of Interaction

Job's method of continuous variation [27] was used for determining the molar ratio of OMZ to each of the two acceptors employed in this investigation. These ratios were found to be 1 : 1 in both cases. This indicates that only one site participated in the formation of the CT complex and a univalent charged species is the possible site of the CT process.

### 3.4. Spectroscopic Investigations for the Structure of the CT Complexes

#### 3.4.1. IR Studies

The structures of OMZ-acceptors complexes were investigated by the IR spectroscopic technique. The majority of IR measurements on the complexes have been concerned with shifts in the vibration frequencies in the donor, acceptor and/or both. The IR spectra of the complexes showed differences compared with those of OMZ and DDQ. These differences have been used to distinguish between weak CT complexes and the products of electron transfer or proton-transfer reactions [24]. As compared to the stretching vibration frequencies of C≡N and C=O bands in the IR spectrum of DDQ (2233, 1678 cm^−1^), a bathochromic shift was observed for these two bands in the IR spectrum of the OMZ-DDQ complex (2227, 1654 cm^−1^). Such a bathochromic shift in these two bands could be indicative for a higher charge density on their corresponding functional groups [21]. These shifts were used as evidence for the formation of CT complexes. The iodine acceptor is IR inactive. Therefore, I compared the IR spectrum of pure OMZ and its corresponding CT complexes with the investigated acceptors. The results indicated that the stretching vibrations of –NH-, C=N, C=C, and S=O bands (3447, 1627, 1472, 1077 cm^−1^, resp.) were bathochromic shifted to lower frequencies (3434, 1627, 1432, 1026 cm^−1^ and 3422, 1618, 1452, 1018 cm^−1^ for OMZ-iodine and OMZ-DDQ CT complexes, resp.).

#### 3.4.2. ^1^H-NMR Studies

In the ^1^H-NMR spectra of the complexes, generally, the protons of the donor are shifted to a lower field [19, 22, 23]. The ^1^H-NMR spectra of the complexes of OMZ with different acceptors were recorded in d_6_-DMSO and compared with the spectrum of the free drug. The 3-methyl, 5-methyl, 4-methoxy protons and the aromatic proton in the position number 2 on the pyridine ring (*δ* = 2.2, 2.2, 3.7 and 8.2 ppm, resp.) were not affected indicating that pyridine ring might not contribute in the electron donation. The protons of CH_2_ attached to the sulphur atom (*δ* = 4.7) were obviously shifted (Δ*δ* = 0.2–0.5 ppm) for both iodine and DDQ complexes. As well, the 5-methoxy protons of benzimidzole ring (*δ* = 3.8 ppm) were obviously downfield shifted (Δ*δ* = 0.2–0.4 ppm). The aromatic protons of C4, C6, and C7 of benzimidazole ring (Δ*δ* =  6.9, 7.1 and 7.5 ppm, resp.) were obviously downfield shifted (Δ*δ* = 0.2–0.5 ppm) for both iodine and DDQ complexes. These results suggested that the electron-donating site in OMZ is close to the aromatic protons of benzimidazole, most probably the imidzole moiety of OMZ. These data, together with above-mentioned IR data, confirmed the CT complex formation between OMZ and both acceptors.

### 3.5. Molecular Modeling for the Charge-Transfer Complexes

**Figure 4:**
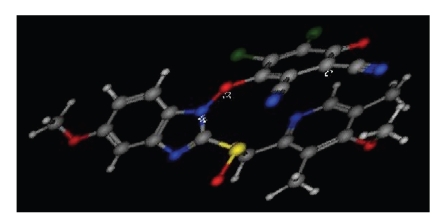
The most energy-minimized conformation of OMZ-DDQ charge-transfer complex.

Molecular modeling for the CT complexes was carried out using the MOPAC package in the CHEM 3D Ultra, version 9.0 (ChemOffice software, CambridgeSoft Corporation, Cambridge, MA, USA) implemented with molecular dynamics computations software (MM2). OMZ and DDQ were energy-minimized alone and both together to obtain the most energyminimized conformation of OMZ CT complexes, the minimum energy of the complex was 130.673 kcal mol^−1^. Figure 4 shows the most energy-minimized conformation of OMZ-DDQ complex. Furthermore, the total charges on all nitrogen atoms were calculated. It was found that DDQ attacks OMZ at the area of benzimidazole moiety. It is acceptable that certain electron density was required for achievement of a successful electron transfer [24]. Comparing the electron density on all nitrogen atoms indicated that the benzimidazole nitrogen in the para-position of OCH_3_-group has the highest density (−0.323) as compared to the other benzimidazole nitrogen (−0.133) and the pyridine nitrogen (−0.223) making it more likely to donate its electrons to the acceptor.

### 3.6. Mechanism of the Studied CT Reactions

**Scheme 3:**
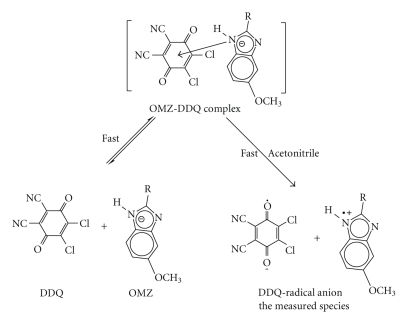


The spectroscopic results confirmed that only one site is possible for the formation of the CT complex. This site, taking into account the molecular modeling for the CT complexes, was postulated to be the benzimidazole moiety of OMZ. Therefore, the plausible mechanism for the reaction of OMZ with the investigated acceptors, taking DDQ as example, was postulated as shown in Scheme 3.

### 3.7. Kinetic Studies

#### 3.7.1. Order of the CT Complex Reactions

Under the described optimum conditions, the absorbance-time curves for the reaction of OMZ with both iodine and DDQ were generated (Figure 3). The initial rates of the reactions were determined from the slope tangents of the absorption-time curves. The order of the reaction with respect to the acceptors was determined by studying the reaction at different concentrations of the acceptors with fixed concentration of OMZ. The plot of the initial rate, *dA*/*dt*, against the initial concentrations of the acceptors was linear indicating that the initial order of the reaction with respect to the acceptors was ≈1. As well, the order with respect to OMZ was evaluated from the measurement of the rates of the CT reactions at different concentrations of OMZ using a fixed concentration of the acceptors, which was found to be ≈1, too. However, under the optimized experimental conditions, the concentration of OMZ was determined using relative excess amount of the acceptors. Therefore, pseudo-first order conditions were obtained and the initial rates of the CT reactions were found to obey the following equation [33]:

(1) $$V=\frac{\Delta A}{\Delta t}=K^{\prime }C^{n},$$

where *V* is the initial reaction rate, *A* is the absorbance, *t* is the measuring time, *K*′ is the pseudo-first-order rate constant, *C* is the molar concentration of OMZ, and *n* is the order of the reaction. The logarithmic form of the above equation is written as follows:

(2) $$\text{Log}\;\;V=\log\;\;\frac{\Delta A}{\Delta t}=\log\;\;\;K^{\prime }+n\;\log\;\;C.$$

The order of the CT reactions was obtained from the slopes (*n*) of Log *V* (at different OMZ concentrations) versus log *C*. The results obtained in Table 2 indicated that the value of (*n*) was 0.87 and 0.99 (≈1) for iodine and DDQ reactions, respectively. This proved that the order of the studied CT reactions was first order. The rate constants (*k*) were calculated from the slopes of the plots of log *At* versus time, where *At* represent the absorbance of the formed complexes at time *t*. Table 2 indicates the values of the specific rate constants of the CT reaction of OMZ with both iodine and DDQ.

#### 3.7.2. Activation Energy and Entropy of Activation of the CT Complexes

The activation energy, the minimum kinetic energy a molecule must possess in order to undergo reaction, can be determined from Arrhenius equation [34]:

(3) $$V=k=Ae^{-\text{Ea}/RT},$$

where *k* is the specific reaction rate, *A* is a constant known as Arrhenius frequency factor, Ea is the activation energy, *T* is the absolute temperature (273+°C), and *R* is the gas constant (1.987 cal mol^−1^°C). The logarithmic form of the above equation is written as follows:

(4) $$\text{Log}\;\;k=\log\;\;\;A-\frac{\text{Ea}}{2.303}RT.$$

The activation energy of the kinetic reaction of OMZ with the investigated acceptors was determined by studying the CT reactions at different temperatures: 25, 40, 60, 70, and 80°C using fixed concentrations of OMZ and the acceptors. The absorption-time curves at these temperatures were constructed to determine the initial rates, then plotting log *k* versus 1/*T* to determine the slope (–Ea/2.303*R*) and the intercept (log *A*) of the line. The obtained activation energies of the investigated complexes were shown in Table 2. The negative sign indicated that the CT complex formation decreased with increase in the temperature. Furthermore, the change in the entropy of activation of the transition state of the complexes was determined using the following equation [34]:

(5) $$A=\left(\frac{RT}{Nh}\right)\cdot e^{∆S^{*}/R},$$

where *A* is the Arrhenius frequency factor, *T* is the absolute temperature, *R* is the gas constant, *N* is Avogadro's number (6.02214179 × 10^23^ mol^−1^), *h* is Planck's constant (6.626 × 10^−34^ J · s), and ΔS* is the change in the entropy of activation (cal mol^−1^_ _°C^−1^). The obtained large negative entropies of activation of the complexes (Table 2) support the formation of more polar transition state in the polar solvent.

#### 3.7.3. Association Constant and Standard Free Energy Change of the CT Complexes

The association constant was evaluated at the corresponding *λ*_max_for each OMZ–acceptor complex using the Benesi-Hildebrand equation [35]:

(6) $$\frac{\left[A_{{}^{\circ}}\right]}{A^{\text{AD}}}=\frac{1}{\varepsilon^{\text{AD}}}+\frac{1}{K_{c}^{\text{AD}}\cdot \varepsilon^{\text{AD}}}\times \frac{1}{\left[D_{{}^{\circ}}\right]},$$

where [*A*_°_] is the concentration of the acceptor; [*D*_°_] is the concentration of the donor; *A* ^AD^ is the absorbance of the complex formed at the specific wavelength; *ε* ^AD^, the molar absorptivity of the complex formed at the specific wavelength; *K*_*c*_^AD^ is the association constant of the complex (l mol^−1^). On plotting the values [*A*_°_]/*A* ^AD^ versus l/[*D*_°_], straight lines were obtained, from which the association constant, correlation coefficient, and the molar absorptivity of OMZ–acceptor complexes were calculated (Table 2). 

The standard free energy change of the complex is related to the association constant by the following equation [34]:

(7) $$\Delta G^{0}=-2.303\;RT\;\log\;\;\;K_{c},$$

where Δ*G* ^0^ is the standard free energy change of the complex; *R* is the gas constant; *T* is the absolute temperature in Kelvin (273+°C); *K*_*c*_ is the association constant of OMZ-acceptor complex (l mol^−1^). As compared to OMZ-iodine complex, the association constant of OMZ-DDQ complex was of much lower value (common feature for CT complexes with *π*-acceptors) due to the dissociation of the original donor-acceptor complex to the radical anion [24]. However, the observed high value for the association constant of the CT complex with iodine suggests that the formed OMZ-iodine complex is of a strong type [21]. Out of the many applications of CT complexes, the determination of the association constant of the CT complexes of drugs with iodine could determine the antithyroid activities of drugs [26]. Therefore, the determination of the association constant of OMZ-I_2_ CT complexes (21.50 × 10^3^ l mol^−1^) could determine the potential iatrogenic antithyroid action of OMZ, and could represent a further tool for the evaluation of drug safety. This would be particularly valuable for prevention of thyroid dysfunction in the neonate [26].

### 3.8. Validation of the Proposed Kinetic Methods

#### 3.8.1. Linearity and Limits of Detection

**(1) Initial Rate Method**

Under the above described optimum conditions, summarized in Table 1, the initial rates of the CT reaction of OMZ with the acceptors would follow a pseudo-first-order kinetic and were found to obey the following equation:

(8) $$\text{Log}\;\;V=\log\;\;\frac{\Delta A}{\Delta t}=\log\;\;K^{\prime }+n\log\;\;C.$$

Regression analysis using the method of least square was performed to evaluate the slope, intercept, and correlation coefficient. The analytical parameters and results of regression analysis are given in Table 3. The limits of detection (LOD) were calculated and found to be 0.24 and 0.41 × 10^−6^ mol (0.08 and 0.14 *μ*g mL^−1^), whereas the limits of quantification (LOQ) were 0.73 and 1.22 × 10^−6^ mol (0.25 and 0.42 *μ*g mL^−1^) for both iodine and DDQ methods, respectively. These low values confirmed the good sensitivity of the initial rate method and consequently its capability to determine OMZ in the linear range of 0.73–8.70 × 10^−6^ mol (0.25–3.00 *μ*g mL^−1^) and 1.45–72.4 × 10^−6^ mol (0.5–25.00 *μ*g mL^−1^) for both iodine and DDQ methods, respectively.

**(2) Fixed Time Method**

In this method, the absorbance of the reaction solutions containing varying amounts of OMZ was measured at a preselected fixed time. Calibration plots of absorbance versus the concentrations of OMZ were established at fixed periods of time for the reactions (Table 4). The regression equations, correlation coefficients, and the limits of detection and quantification are given in Table 4. The lowest limits of detection and quantification were obtained with fixed times of 45 minutes (for iodine) and of 4 and 5 minutes (for DDQ) methods, respectively. However, the fixed times of 5 minutes (for iodine) and of 1 minute (for DDQ) methods showed wider dynamic range for quantification. Therefore, on the basis of wider dynamic range and less time of analysis, the fixed time of 5 and 1 minute for iodine and DDQ methods, respectively, was recommended for the determination of OMZ by the fixed time method, if the sensitivity is not required (otherwise, fixed time of 45 and 5 minutes is better for both methods, resp.).

Out of the many advantages of the kinetic spectrophotometric methods, one important advantage is that the long reaction time could be overcome by applying the initial rate method. This was the case in our proposed iodine method. Therefore, the initial rate was preferred than the fixed time with respect to iodine method. However, the fixed time is preferred than the initial rate with respect to DDQ method due to its short reaction time (5 minutes).

#### 3.8.2. Precision and Accuracy

The precision and accuracy were tested by applying the proposed methods using the same experimental conditions as described under the general analytical procedure. 

The precision of the proposed methods was determined [36] at three concentration levels of OMZ (0.5, 1.5, and 3.0 *μ*g mL^−1^ for iodine and 2.0, 10.0, and 20.0 *μ*g mL^−1^ for DDQ methods). This was performed by determination of five replicate samples of each concentration by both the initial rate and fixed time methods. The results revealed that the relative standard deviations (RSDs) of the values did not exceed 1.25%.

The accuracy of the proposed methods was also evaluated using the same (above-mentioned) concentrations of OMZ. Table 5 showed the recovery results (98.50%–100.80%) with RSD not more than 1.95%, proving the high accuracy of the proposed methods. This high level of the precision and accuracy indicated the suitability of the proposed methods for the quality control analysis of OMZ in its pharmaceutical preparations.

#### 3.8.3. Specificity and Interference

The proposed kinetic methods have the advantage of that all measurements are performed in the visible region, away from the near UV absorbing interfering substances that might be coextracted from OMZ-containing dosage forms. The potential interferences from the excipients in the dosage forms were studied. Samples were prepared by mixing known amount (20 mg) of OMZ with various amounts of the common excipients such as starch, glucose, lactose, acacia, and magnesium stearate. The results (Table 6) revealed that no interference was observed from any of these excipients with the proposed methods.

#### 3.8.4. Ruggedness and Robustness

The ruggedness was tested by applying the proposed methods to the assay of OMZ using the same operational conditions but using two different instruments at two different laboratories and different elapsed time. Results obtained from lab-to-lab and day-to-day variations were reproducible as RSD did not exceed 3%. 

Robustness of the proposed procedures was examined by evaluating the influence of small variation in the concentration of acceptor reagents (within ± 5%) or in the reaction temperature (25 ± 2°C) on the analytical performance of the proposed methods. In these experiments, one experimental variable was changed, whereas the others were kept unchanged, and the recovery percentage was calculated each time. It was found that neither the temperature (23, 25, and 27°C were tested) nor the reagents concentration (1.9, 2.0, and 2.1 mg mL^−1^ of iodine and 3.8, 4.0, and 4.2 mg mL^−1^ of DDQ reagents were tested) significantly affected the results; the recovery percentages were 98.64%–101.53% ± 0.92–1.67. This provided an indication for the reliability of the proposed methods during routine work.

### 3.9. Application of the Proposed Methods to the Analysis of Pharmaceutical Preparations

Depending on the obtained validation results, the proposed procedures were found to be suitable for the routine quality control analysis of OMZ. The proposed and the reference methods [12] were applied to the determination of OMZ in its dosage forms. The results obtained by the proposed methods were statistically compared with those obtained by the reference method. The recovery of the labeled amount was 98.91–100.32 ± 0.94%–1.84% (Table 7). The results of *t*- and *F*-tests revealed that no significant differences were found between the proposed and reference methods at 95% confidence level with respect to precision and accuracy. This proved the applicability of the proposed methods for quality control analysis of OMZ in its pharmaceutical preparations with comparable analytical performance.

## 4. Conclusions

The present work utilized the colored CT complexes of OMZ with both iodine and DDQ in the development of new simple, rapid, sensitive, and accurate kinetic spectrophotometric methods for the analysis of OMZ in dosage forms. The obtained CT complexes have been investigated by UV-VIS spectrophotometry, IR, and ^1^H-NMR spectroscopic techniques, and by computational molecular modeling. A plausible mechanism for these CT reactions based on the spectroscopic study was postulated. The proposed kinetic methods are superior to the previously reported spectrophotometric methods in the term of sensitivity and improved selectivity because the sensitivity of the proposed methods (linear range was as low as 0.1 *μ*g mL^−1^) is higher than that of all reported spectrophotometric methods (the lowest reported linear range was 0.2 *μ*g mL^−1^) for determination of OMZ [3–13]. Furthermore, the measurements of most of the reported spectrophotometric methods were performed at lower wavelengths (at 258–320 nm) which may be influenced by the excipients of the dosage forms in contrast to the proposed kinetic methods [5–10]. Moreover, some of the reported colorimetric methods are time consuming [4, 9], and others have been proved to be inaccurate due to matrix interference [3].

## Figures and Tables

**Table 1:** Optimum conditions for the charge-transfer reaction of OMZ with both iodine and DDQ reagents.

Conditions	Iodine method	DDQ method
Acceptor conc. (mg mL^−1^)	2.0	4.0
Solvent	1,2-Dichloroethane	Acetonitrile
Reaction time (min)	45	5
Temperature (°C)	25 ± 2	25 ± 2
*λ*_max_ (nm)	362	418

**Table 2:** Kinetic parameters obtained from Benesi-Hildebrand, Arrhenius, and first-order linear plots of OMZ-acceptor CT complexes at the specific *λ*_max  _ and reaction temperature.

Kinetic parameters	Iodine method	DDQ method
Correlation coefficient (*r*)	0.9995	0.9999
Order of CT reaction (*n*)	0.87	0.99
Specific rate constant, s^−1^(*k*) × 10^4^	5.45	56.18
Activation energy, kcal mol^−1^ (Ea)^♠^	−1.54	−1.95
Activation entropy change, kcal mol^−1^°C (Δ*S*°)	−69.96	−66.44
Molar absorptivity, l · mol^−1^ cm^−1^(*ε*) × 10^3^	21.12	6.89
Standard free energy change, kcal mol^−1^(Δ*G*°)	−5.91	−4.80
Association constant, l · mol^−1^(*K*_*c*_) × 10^3^	21.50	3.33

^♠^ : Negative sign of Ea indicates that the CT complex is decomposed by increasing temperature.

**Table 3:** Analytical parameters for the proposed kinetic initial rate methods for determination of OMZ.

Analytical parameters	Iodine method	DDQ method
Linear range (mol)	0.73–8.70 × 10^−6^	1.45–72.4 × 10^−6^
	(0.25–3.00)^a^	(0.50–25.00)
Least square equation		
(Log *V* = log *K*′+*n*log *C*)^b^		
* *Intercept (log *K*′) ± SD	2.68 ± 0.10	3.36 ± 0.03
* *Slope (*n*) ±SD	0.87 ± 0.02	0.99 ± 0.01
* *Correlation coefficient (*r*)	0.9990	0.9996
LOD^c^ (mol)	0.24 × 10^−6^ (0.08)	0.41 × 10^−6^ (0.14)
LOQ^c^ (mol)	0.73 × 10^−6^ (0.25)	1.22 × 10^−6^ (0.42)

^a^ Figures in parenthesis are the quantities in *μ*g mL^−1^.

^b^ *V* is the reaction rate, *K*′ is the conditional rate constant, *n* is the order of reaction, and *C* is the molar concentration of OMZ.

^c^ To simplify the calculation of both LOD and LOQ, the simple nonlogarithmic equations [*V * = 0.001(±0.00015) + 2093(±45)*C*] and [*V * = 0.002(±0.00027) + 2391(±39)*C*] were used for both iodine and DDQ methods, respectively, where *V* is the reaction rate and *C* is the molar concentration of OMZ.

**Table 4:** Analytical parameters for the proposed fixed time methods for determination of OMZ.

Method	Time	Range	*R*	Slope	Intercept	LOD	LOQ
	(min)	(*μ*g mL^−1^)		(b) ± SD	(a) ± SD	(*μ*g mL^−1^)	(*μ*g mL^−1^)
Iodine	5	1.50–12.00	0.9915	0.0751 ± 0.0049	0.0226 ± 0.0095	0.42	1.27
	15	0.50–6.00	0.9955	0.1589 ± 0.0017	0.0060 ± 0.0073	0.15	0.46
	25	0.40–5.00	0.9992	0.2198 ± 0.0044	0.0064 ± 0.0085	0.13	0.39
	35	0.30–4.00	0.9995	0.2649 ± 0.0042	0.0323 ± 0.0061	0.08	0.23
	45	0.10–3.00	0.9990	0.3069 ± 0.0067	0.0693 ± 0.0032	0.03	0.10

DDQ	1	10.00–120.00	0.9982	0.0065 ± 0.0002	0.0013 ± 0.0065	3.28	9.94
	2	5.00–60.00	0.9992	0.0155 ± 0.0001	0.0097 ± 0.0028	0.59	1.80
	3	3.00–40.00	0.9993	0.0249 ± 0.0006	0.0246 ± 0.0036	0.48	1.45
	4	2.50–30.00	0.9992	0.0320 ± 0.0007	0.0305 ± 0.0017	0.18	0.53
	5	0.60–25.00	0.9998	0.0366 ± 0.0004	0.0299 ± 0.0021	0.19	0.57

**Table 5:** Evaluation of the accuracy of the proposed initial rate and fixed time methods for determination of OMZ.

Method	Amount taken (*μ*g mL^−1^)	Recovery (% ± RSD)^a^	
		Initial rate method	Fixed time method
Iodine	0.50	99.50 ± 1.95	100.40 ± 0.94
	1.50	98.80 ± 1.08	99.80 ± 0.25
	3.00	99.70 ± 1.55	98.50 ± 0.42

DDQ	2.00	98.60 ± 1.45	99.60 ± 1.84
	10.00	98.90 ± 0.95	100.80 ± 0.75
	20.00	100.30 ± 0.75	100.20 ± 1.25

^a^ Recovery was calculated as the amount found/amount taken ×100. Values are mean ± RSD for five determinations.

**Table 6:** Determination of OMZ in presence of common excipients by the proposed fixed time methods.

Ingredient	Recovery (% ± SD)^a^	
	Iodine method	DDQ method
Starch (50)^b^	99.26 ± 0.39	98.86 ± 0.66
Glucose (10)	99.42 ± 1.02	99.33 ± 1.53
Lactose (10)	100.49 ± 1.35	101.11 ± 1.45
Acacia (10)	101.02 ± 0.75	100.85 ± 0.64
MS^c^ (10)	99.31 ± 0.96	98.94 ± 0.75

Average ± SD	99.90 ± 0.80	99.82 ± 1.08
Pool SD	0.89	1.01

^a^ Values are mean of three determinations.

^b^ Figures in parenthesis are the amounts in mg added per 20 mg of OMZ.

^c^ MS  =  Magnesium stearate.

**Table 7:** Determination of OMZ in its dosage forms by the proposed methods.

Dosage form	Label claim (% ± SD)^a^				
	Initial rate method		Fixed-time method		Reference^c^ method
	Iodine	DDQ	Iodine	DDQ	

Gastrazole capsules	99.82 ± 1.65	98.91 ± 1.78	100.24 ± 1.84	99.20 ± 1.53	100.40 ± 1.34
* t*-value^b^	0.63	1.51	0.2	1.32	
* F*-value^b^	1.52	1.76	1.89	1.30	

Losec tablets	99.41 ± 1.24	99.70 ± 1.13	100.32 ± 0.94	99.93 ± 1.13	100.90 ± 1.18
* t*-value^b^	1.96	1.64	0.14	0.64	
* F*-value^b^	1.10	1.09	0.63	0.92	

^a^ Values are mean of five determinations.

^b^ The tabulated values of *t* and *F* at 95% confidence limit are 2.78 and 6.39, respectively.

^c^ Reference [12].
